# The double-edged sword of generative AI in dermatology: a multi-component cross-sectional study on physician burnout, patient satisfaction, and communication quality

**DOI:** 10.3389/fmed.2026.1875075

**Published:** 2026-07-08

**Authors:** Yunpeng Wei, Hong Xu, Yuan Hu

**Affiliations:** 1 Intensive Care Unit, The First Affiliated Hospital of Zhengzhou University, Zhengzhou, Henan, China; 2 Department of Dermatology, The First Affiliated Hospital of Zhengzhou University, Zhengzhou, Henan, China; 3 Department of Dermatology, Suining Central Hospital, Suining, Sichuan, China

**Keywords:** dermatology, generative artificial intelligence, occupational burnout, patient satisfaction, physician-patient communication, standardized case assessment

## Abstract

**Background:**

Generative artificial intelligence (GenAI), particularly large language models (LLMs), is rapidly integrating into clinical settings. However, its net effect on dermatological practice remains poorly defined. This study investigates the dual impact of GenAI on clinician–patient communication using a multi-component data approach.

**Methods:**

We conducted an exploratory multi-component cross-sectional study from February 2025 to January 2026 in the dermatology departments of two tertiary hospitals in China. This study included physician surveys (*n* = 25), outpatient questionnaires (*n* = 60), and standardized case assessments. For the standardized component, 20 physicians completed two cases under randomized no-AI and AI-assisted pre-consultation preparation sequences, yielding 80 assessment records. The AI-assisted condition used DeepSeek-R1 for 5 min of pre-consultation preparation before a 10-min standardized patient encounter.

**Results:**

Physician GenAI use frequency was associated with lower emotional exhaustion (r_s = −0.692, *p* = 0.002) and higher communication self-efficacy (r_s = 0.848, *p* < 0.001). In the adjusted physician model, GenAI use frequency was associated with higher PCSES scores (*B* = 3.204, 95% CI 2.263 to 4.145), and this association was maintained in leave-one-out analyses (*B* range 3.010–3.374), a parsimonious model (*B* = 3.151), and a model excluding influential observations (*B* = 3.055). Patient GenAI users reported higher communication satisfaction than non-users (18.46 ± 2.48 vs. 15.17 ± 2.43, *p* < 0.001). In standardized cases, the AI-assisted condition was associated with higher estimated marginal scores for information gathering (difference 3.175, 95% CI 0.605 to 5.745), information giving (5.675, 95% CI 3.432 to 7.918), structural efficiency (5.575, 95% CI 3.751 to 7.399), and total score (2.490, 95% CI 1.374 to 3.606). Humanistic care showed a reduction in the AI-assisted condition in the primary mixed-effects model; however, this difference did not reach statistical significance. Sensitivity analyses using fully adjusted mixed-effects models accounting for period, condition order, and case order indicated a consistent negative effect of AI assistance on humanistic care (*B* = −3.414, 95% CI −5.723 to −1.105), indicating the sensitivity of this outcome to model specification, with a consistent negative effect observed in fully adjusted models.

**Conclusion:**

These exploratory findings suggest that GenAI-assisted preparation was associated with stronger information organization and communicative efficiency in dermatology, while not automatically improving empathic or humanistic communication. GenAI should therefore be positioned as a supervised supportive tool rather than as a replacement for clinical judgment or relational care.

## Introduction

Since the advent of ChatGPT, generative artificial intelligence (GenAI) has rapidly spread throughout the healthcare industry, with applications spanning patient education, automated messaging, and clinical documentation (1, 2). Dermatology is uniquely positioned as a representative specialty for observing the impact of such technologies on physician–patient communication, given its visual diagnostic nature, high outpatient turnover, and the intensive requirement for patient education (3, 4).

Current research in dermatology consistently indicates that both clinicians and patients favor a “physician-AI collaboration” model over autonomous AI systems for diagnosis or communication (5, 6). From the provider’s perspective, primary interests focus on patient education, administrative support, and the alleviation of repetitive communicative burdens (7). For patients, the appeal of GenAI lies in its accessibility, anonymity, and its utility in pre-consultation preparation (8, 9). Despite these benefits, concerns regarding misinformation, “hallucinations,” privacy risks, and the potential dehumanization of the clinical relationship remain central barriers to implementation (10).

Crucially, existing evidence has largely remained fragmented, focusing independently on physician attitudes, patient acceptance, or the technical accuracy of model responses (11–13). For clinical integration, simply determining whether AI is “useful” is insufficient. A more critical inquiry involves identifying which specific dimensions of communication are enhanced by GenAI and which elements of the therapeutic alliance may be inadvertently compromised (14–16).

To address these gaps, this study integrated physician, patient, and standardized case components to examine the interplay between GenAI use, physician wellbeing, patient outcomes, and observable communication performance. These domains represent complementary levels of clinical communication: Physician burnout and communication self-efficacy define clinician-side workload and confidence; patient satisfaction and perceived empathy define patient-experienced communication quality; and standardized case performance provides a more controlled assessment of communication behaviors. By synthesizing these three components, we aimed to clarify which dimensions of dermatological communication may benefit from GenAI and which may remain vulnerable to depersonalization.

## Methods

### Study design

**Figure 1:**
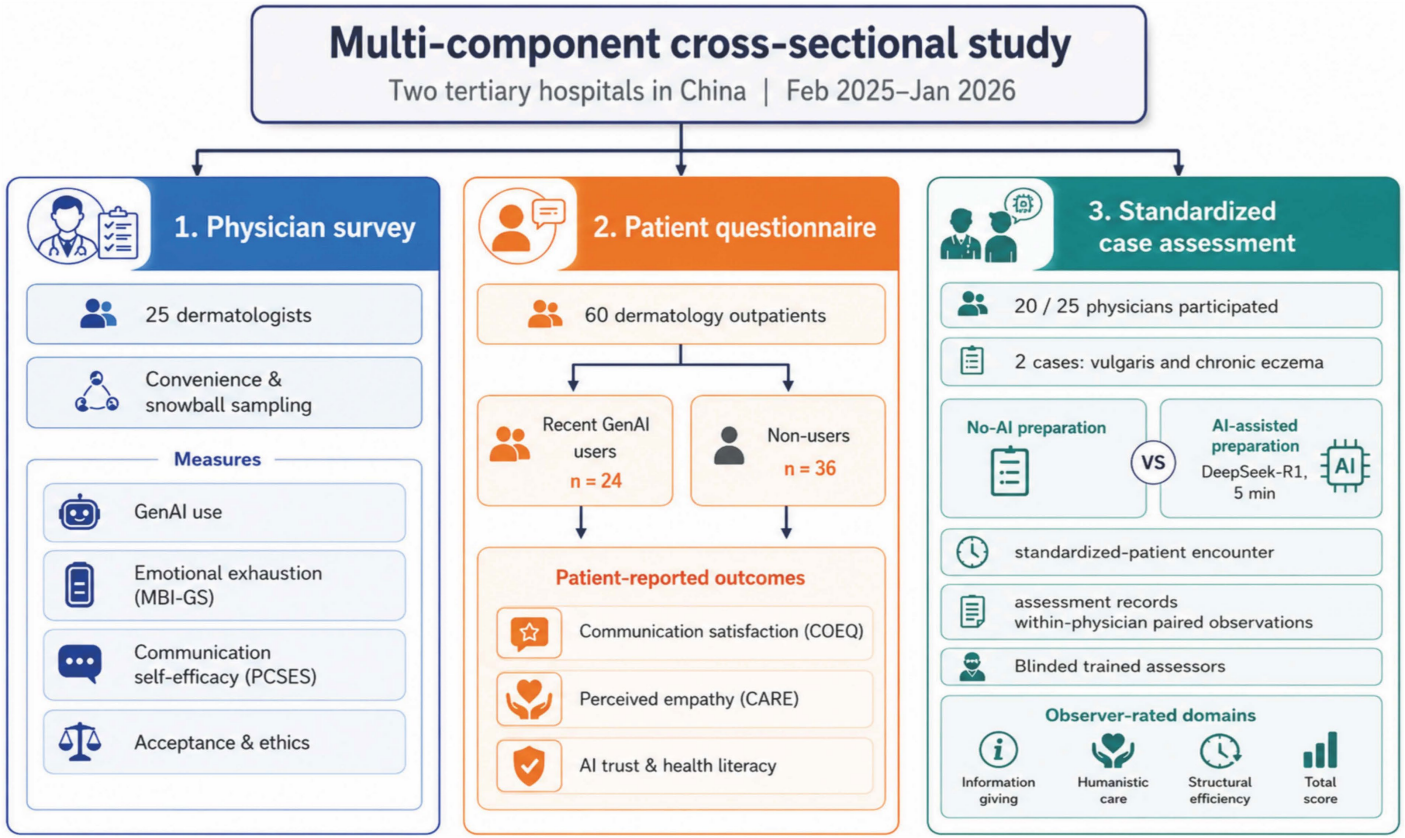
Study flow and analytical framework.

This exploratory multi-component cross-sectional study integrated physician surveys, patient questionnaires, and standardized case assessments across the dermatology departments at the First Affiliated Hospital of Zhengzhou University and Suining Central Hospital. Data were collected from February 2025 to January 2026. The study flow and analytical framework are summarized in Figure 1. The physician component included 25 dermatologists, the patient component included 60 outpatients (24 recent GenAI users and 36 non-users), and the standardized case component included 20 physicians who completed two cases under non-AI-assisted and AI-assisted preparation conditions, yielding 80 records and 40 within-physician paired observations.

This multi-component study was conducted at two tertiary hospitals in China from February 2025 to January 2026. The physician component included a survey of 25 dermatologists; the patient component included 60 dermatology outpatients, comprising 24 recent GenAI users and 36 non-users; and the standardized case component included 20 physicians who completed two clinical cases under no-AI and AI-assisted pre-consultation preparation conditions, yielding 80 records and 40 within-physician paired observations. The three components were analyzed using complementary correlation, regression, repeated-measures, mixed-effects, and inter-rater reliability methods to integrate physician-, patient-, and observer-rated communication outcomes.

### Study participants

Physicians were recruited using convenience sampling combined with snowball sampling. Recruitment information was distributed through departmental work groups, and physicians who expressed willingness to participate were screened for eligibility. The inclusion criteria were as follows: active clinical dermatologists who had worked continuously in the dermatology department of either participating hospital for at least 1 year, who had outpatient duties of at least 2 half-day sessions per week, and who had voluntarily signed informed consent. The exclusion criteria were as follows: visiting doctors, standardized residency trainees, interns, or physicians who had participated in the research team’s preliminary pilot testing.

Patients were recruited in the outpatient waiting area by trained graduate students. The study purpose was explained to potentially eligible patients, and those who agreed completed the questionnaire after the consultation. The inclusion criteria were as follows: patients who were aged 18–65 years, patients who had a first or follow-up dermatology visit during the study period, and patients who had basic reading and writing ability sufficient to complete the questionnaire independently. The exclusion criteria were as follows: patients with a history of cognitive impairment or psychiatric disease, with severe visual or hearing impairment affecting communication, or with current participation in another interventional clinical trial.

The physician cohort consisted of 25 active dermatologists (mean age: 37.72 ± 7.13 years; mean professional experience: 8.16 ± 5.68 years). The patient cohort included 60 outpatients. They were categorized into those who had used GenAI for skin health information within the past 4 weeks (*n* = 24) and those who had not used GenAI (*n* = 36). Overall, 20 of the 25 physicians voluntarily participated in the second-stage standardized case assessment. The five physicians who did not participate in the second stage were unable to attend because of work schedule conflicts.

Sample size was primarily determined by feasibility because this was an exploratory cross-sectional study. No formal *a priori* sample size calculation was performed for the physician cohort; the target was to recruit approximately 25–30 dermatologists. The final physician sample included only three participants in the frequent use category, so subgroup-specific estimates should be interpreted cautiously. The patient sample size was also feasibility-based. Given the exploratory design, no formal *a priori* power calculation was used to determine the final sample size.

### Variables, measurements, and GenAI exposure definitions

Physician-level variables included GenAI use frequency, the emotional exhaustion subscale of the Maslach Burnout Inventory-General Survey (MBI-GS), communication self-efficacy, technology acceptance indicators, and AI-related ethical concerns. Physician GenAI use frequency was assessed using the following item: “In your recent dermatology outpatient work, how often have you used generative AI tools (such as DeepSeek, ChatGPT, Doubao, Qianwen, Kimi, or similar large language model tools) for patient education, explanation drafting, medical information organization, or communication preparation?” Responses were coded on a four-point scale: 1 = never, 2 = occasionally (once per week), 3 = sometimes (2–3 times/week), and 4 = frequently/often (>3 times/week).

The patient’s recent GenAI use was assessed using the following item: “During the past 4 weeks, have you used any generative AI or large language model tool (for example, DeepSeek, ChatGPT, Doubao, Qianwen, Kimi, Gemini, or similar tools) to obtain information related to your skin condition or skin health?” Patients answering “yes” were further asked to report the main platform, primary purpose of use, and approximate frequency of use. These questionnaire items were developed with reference to prior survey studies on patient use of AI and large language models for health information seeking and were reviewed by the research team for dermatology relevance.

For patient-side GenAI utilization, available descriptors included platform type, primary use purpose, and use frequency. Platforms included Doubao, DeepSeek, Qianwen, Kimi, and ChatGPT/Gemini. The main use purposes were grouped as symptom inquiry, treatment consultation, and second opinion seeking. Since patient-side platform and use pattern heterogeneity may influence the association with communication outcomes, these variables were treated as descriptive contextual information rather than causal predictors.

### Standardized case procedure

In the standardized case component, each of the 20 participating physicians completed four simulated encounters on the same day, covering all four condition-by-case combinations: no-AI/acne vulgaris, AI-assisted/acne vulgaris, no-AI/chronic eczema, and AI-assisted/chronic eczema. The encounter order was randomized at the physician level using a pre-generated random sequence to balance condition and case order across participants. Therefore, both the preparation condition order and clinical case order varied across physicians. The recorded sequences confirmed that each physician completed all four unique condition-by-case combinations exactly once. Encounters were completed consecutively with operational turnover intervals between encounters. In AI-assisted periods, physicians used DeepSeek-R1 during a 5-min pre-consultation preparation window and were allowed to retain electronic notes generated during preparation; GenAI was not used during the 10-min face-to-face standardized patient encounter. Assessors were blinded to the preparation condition and did not receive sequence or AI-use information.

Standardized case performance was evaluated by three trained independent assessors using blinded scoring procedures. The assessor panel included an attending dermatologist with additional training in medical humanities, an associate professor in medical communication with a public health background, and a senior dermatology resident who had completed dedicated physician–patient communication training. Each record was independently scored by two assessors and averaged; when scores differed by more than 15 points, the third assessor determined the final score.

The five scoring domains were information gathering, information provision, humanistic care, structural efficiency, and overall score, each scale ranged from 0 to 100. For the humanistic care domain, scores of 90–100 indicated active recognition of patient emotion, empathic language, emotional support, respect for privacy concerns, and patient-centered pacing; 80–89 indicated basic politeness with relatively passive emotional response; 60–79 indicated mechanical question–answer communication with limited relational engagement; and 0–59 indicated interruption, neglect of patient concerns, coldness, or inappropriate wording.

Inter-rater reliability was assessed using the intraclass correlation coefficient (ICC). During formal scoring, each record was independently evaluated by two assessors, and the mean of the two scores was taken as the final rating. In cases where the absolute discrepancy between the two assessors exceeded 15 points, the record was submitted to a third assessor for review, and the resulting score was used as the final rating. The complete scoring rubric, AI-assisted preparation prompt, and inter-rater reliability results are provided in Supplementary Tables S1–S3, respectively.

### Questionnaire instruments

The physician questionnaire assessed emotional exhaustion using the Maslach Burnout Inventory-General Survey (MBI-GS) emotional exhaustion subscale, communication self-efficacy using the Physician Communication Self-Efficacy Scale (PCSES), technology acceptance using constructs derived from the Unified Theory of Acceptance and Use of Technology (UTAUT), and AI-related ethical concerns using study-specific items. The patient questionnaire assessed eHealth literacy using the eHealth Literacy Scale (eHEALS), communication satisfaction using the communication domain of the Communication Outcomes Evaluation Questionnaire (COEQ), perceived clinician empathy using the Consultation and Relational Empathy (CARE) measure, and trust in AI using study-specific/adapted items. The patient questionnaire assessed eHealth literacy using eHEALS, communication satisfaction using the COEQ communication domain, perceived clinician empathy using CARE, and trust in AI using study-specific/adapted items. Detailed information on each instrument or indicator, including source/adaptation, number of items, score range, and score direction, is provided in Supplementary Table S11.

### Statistical analysis

Continuous variables were presented as means ± standard deviation (SD), and categorical variables were presented as frequencies and percentages. Patient baseline categorical variables were compared using Pearson’s chi-squared tests, with Yates continuity correction for 2 × 2 tables. Relationships between physician-level variables were analyzed using Spearman’s rank correlations with 95% confidence intervals (CIs). For the physician regression model, PCSES communication self-efficacy was used as the dependent variable, and GenAI use frequency was coded as an ordinal four-level predictor. The primary physician model included GenAI use frequency, professional title, years of clinical experience, and hospital affiliation; collinearity was assessed using variance inflation factors (VIFs). Since professional title and years of experience were highly correlated, a parsimonious sensitivity model retained years of experience but omitted professional title. Influence diagnostics for the adjusted physician model included Cook’s distance, leverage, externally studentized residuals, differences in beta coefficients (DFBETAs), leave-one-out refitting, and refitting after excluding observations flagged by prespecified thresholds. Patient-reported continuous outcomes were compared using two-sided independent-samples *t*-tests. A multivariable linear regression model for communication satisfaction was adjusted for age, educational attainment, hospital affiliation, and dermatologic disease category. Standardized case outcomes were analyzed using paired-samples *t*-tests and linear mixed-effects models with fixed effects for AI condition, case type, and their interaction and a random intercept for the physician. Degrees of freedom for LMMs were estimated using the Satterthwaite approximation, and estimated marginal means were used to calculate the overall AI-assisted and no-AI contrast averaged across case type. Additionally, sensitivity LMMs were adjusted for encounter period, condition order, and case order. Inter-rater reliability was estimated using two-way random-effects absolute-agreement intraclass correlation coefficients for single and average measures. All tests were two-sided, with a *p*-value of <0.05 indicating statistical significance. Analyses were performed using Python 3.11.5 and R 4.5.2 with lme4, lmerTest, and emmeans.

## Results

### Baseline characteristics

Among the physician cohort, the distribution of GenAI usage frequency was as follows: “never” (*n* = 2, 8.0%), “occasionally” (*n* = 9, 36.0%), “sometimes” (*n* = 11, 44.0%), and “frequently” (*n* = 3, 12.0%) (Table 1A; Supplementary Table S4).

**Table 1A:** Physician characteristics.

Characteristics	Physicians (*n* = 25)
Age, years	37.72 ± 7.13
Work experience, years	8.16 ± 5.68
Hospital, *n* (%)	—
First Affiliated Hospital of Zhengzhou University	15 (60.0)
Suining Central Hospital	10 (40.0)
Male sex, *n* (%)	12 (48.0)
Professional title, *n* (%)	—
Resident physician	8 (32.0)
Attending physician	10 (40.0)
Associate chief physician	5 (20.0)
Chief physician	2 (8.0)
GenAI use frequency, *n* (%)	—
Never	2 (8.0)
Occasionally	9 (36.0)
Sometimes	11 (44.0)
Often	3 (12.0)

Values are mean ± SD or *n* (%), as appropriate.

Within the patient cohort, GenAI users were younger than non-users (37.9 ± 10.2 vs. 44.1 ± 10.1 years; *p* = 0.024). Educational attainment did not differ significantly between the groups in Table 1B [chi-square(3)=1.493, *p* = 0.684]. GenAI users had numerically higher eHealth literacy scores (23.38 ± 3.87 vs. 21.78 ± 4.34), and no statistically significant differences were observed between the two groups regarding sex, hospital affiliation, or privacy sensitivity. Detailed demographic profiles, disease distributions, and descriptive statistics are presented in Table 1B.

**Table 1B:** Patient characteristics by recent GenAI use.

Characteristics	Users (*n* = 24)	Non-users (*n* = 36)	Statistics	*p*-value
Age, years	37.88 ± 10.21	44.08 ± 10.17	*t*(58) = −2.313	0.024
Male sex, *n* (%)	8 (33.3)	10 (27.8)	*χ*^2^(1) = 0.030	0.863
Hospital, *n* (%)	—	—	—	—
First Affiliated Hospital of Zhengzhou University	16 (66.7)	19 (52.8)	χ^2^(1) = 0.643	0.423
Suining Central Hospital	8 (33.3)	17 (47.2)	—	—
Education, *n* (%)	—	—	—	—
Junior high or below	3 (12.5)	5 (13.9)	χ^2^(3) = 1.493	0.684
High school/technical secondary	11 (45.8)	11 (30.6)	—	—
Bachelor’s degree	6 (25.0)	12 (33.3)	—	—
Postgraduate or above	4 (16.7)	8 (22.2)	—	—
Primary dermatologic diagnosis, *n* (%)	—	—	—	—
Inflammatory dermatoses	13 (54.2)	9 (25.0)	χ^2^(4) = 11.472	0.022
Infectious dermatoses	0 (0.0)	11 (30.6)	—	—
Neoplastic/pigmentary dermatoses	4 (16.7)	6 (16.7)	—	—
Cosmetic-related dermatoses	5 (20.8)	5 (13.9)	—	—
Other	2 (8.3)	5 (13.9)	—	—
Privacy-sensitive concern, *n* (%)	3 (12.5)	5 (13.9)	χ^2^(1) = 0.000	1.000

Recent GenAI use refers to self-reported use for skin health information during the previous 4 weeks. — indicates not applicable.

### Physician perspective: GenAI usage, burnout, and communication self-efficacy

**Figure 2:**
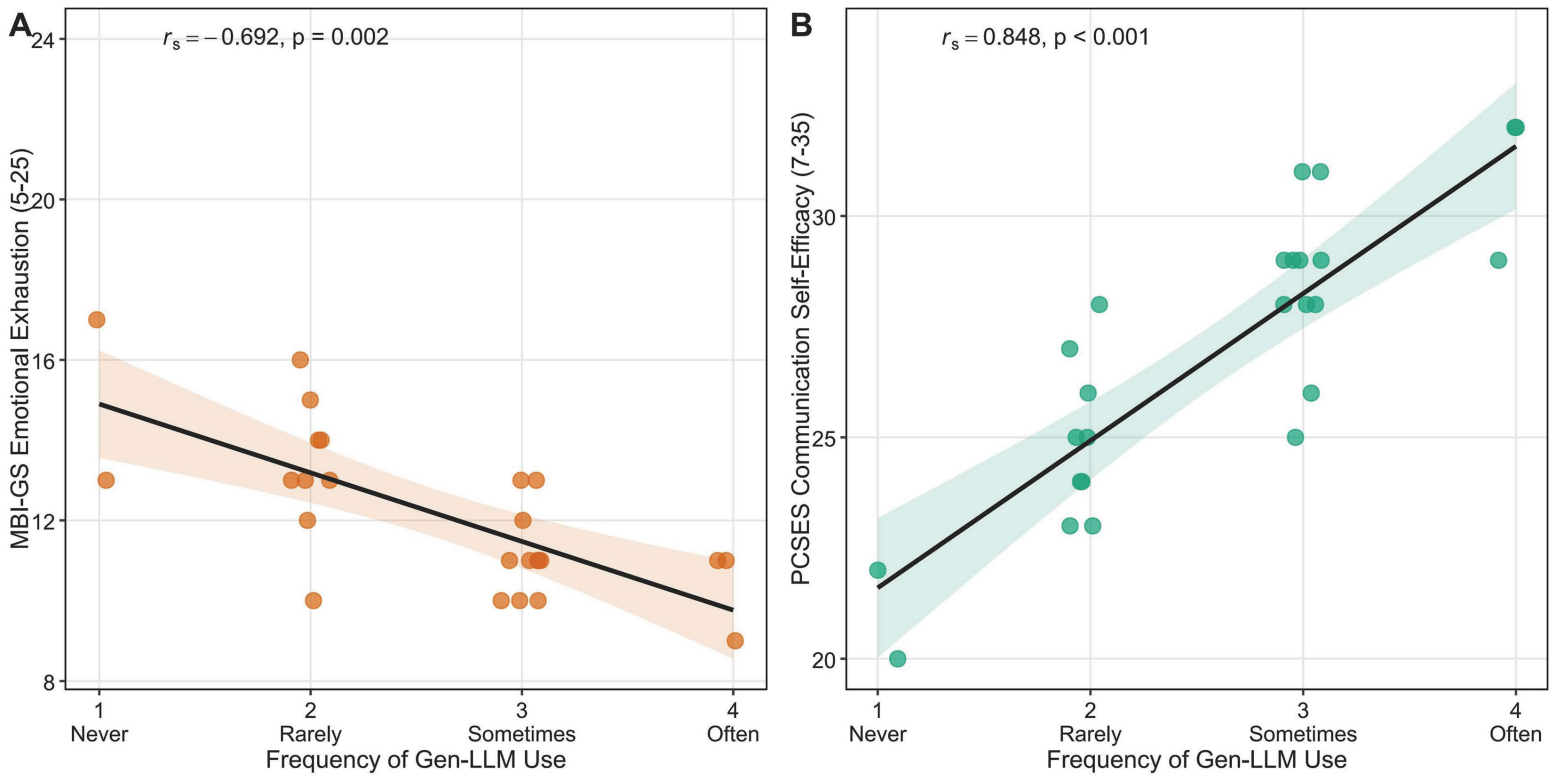
GenAI use frequency was negatively correlated with emotional exhaustion (**A**; r_s = −0.692, *p* = 0.002) and positively correlated with communication self-efficacy (**B**; r_s = 0.848, *p* < 0.001). Individual data points are shown to allow visual assessment of leverage and distribution across the four-point frequency scale (1 = never, 2 = occasionally, 3 = sometimes, 4 = often). Lines represent regression fits with 95% confidence intervals.

Spearman’s correlation analysis revealed that the frequency of GenAI usage among dermatologists was significantly and negatively correlated with the emotional exhaustion subscale of the MBI-GS (r_s = −0.692; 95% CI, −0.866 to −0.369; *p* = 0.002). Conversely, usage frequency showed a strong positive correlation with communication self-efficacy, as measured by the PCSES (r_s = 0.848; 95% CI, 0.671 to 0.935; *p* < 0.001). Furthermore, GenAI usage frequency was positively associated with perceived usefulness under the UTAUT framework (r_s = 0.518, *p* = 0.008). However, no significant correlation was observed between usage frequency and total scores for AI-related ethical concerns (*p* = 0.198) (Table 2; Figure 2; Supplementary Figure S1).

**Table 2:** Physician-side associations and sensitivity analyses.

Variable	Spearman r_s (95% CI)	*p*-value
MBI-GS emotional exhaustion	−0.692 (−0.866 to −0.369)	0.002
PCSES communication self-efficacy	0.848 (0.671 to 0.935)	<0.001
UTAUT perceived usefulness	0.518 (0.155 to 0.758)	0.008
UTAUT perceived ease of use	0.450 (0.066 to 0.717)	0.024
Total AI ethics concern	−0.266 (−0.598 to 0.144)	0.198

Predictor	*B* (SE)	95% CI	*t*	*p*-value
GenAI use frequency	3.204 (0.480)	2.195 to 4.213	6.670	<0.001
Attending physician (vs resident)	1.573 (1.200)	−0.949 to 4.095	1.310	0.207
Associate chief physician (vs resident)	0.488 (2.024)	−3.765 to 4.741	0.241	0.812
Chief physician (vs resident)	3.901 (3.098)	−2.609 to 10.411	1.259	0.224
Work experience, years	−0.097 (0.159)	−0.432 to 0.237	−0.612	0.548
Suining Central Hospital (vs FAHZZU)	0.907 (0.653)	−0.466 to 2.279	1.388	0.182

The primary full model was adjusted for professional title, years of experience, and hospital affiliation. The parsimonious sensitivity model omitted professional title because of its high correlation with years of experience. Detailed VIFs and influence diagnostics are reported in Supplementary Tables S5, S6.

In the primary multivariable model for PCSES communication self-efficacy, GenAI use frequency remained positively associated with PCSES scores after adjusting for professional title, years of clinical experience, and hospital affiliation (*B* = 3.204; SE = 0.480; 95% CI, 2.263 to 4.145; *p* < 0.001). Professional title and years of experience were strongly correlated (Spearman r = 0.920), and VIFs ranged from 1.03 to 7.90, with the highest values observed for professional title and work experience. Therefore, a parsimonious sensitivity model retaining years of experience but omitting professional title was also fitted; the GenAI coefficient remained similar (*B* = 3.151; 95% CI, 2.200 to 4.101; *p* < 0.001) (Table 2; Supplementary Table S5).

Influence diagnostics for the adjusted physician model identified four potentially influential observations using prespecified thresholds for Cook’s distance, leverage, studentized residuals, and DFBETAs. In leave-one-out refitting, the GenAI coefficient remained positive for every excluded physician (*B* range, 3.010–3.374), and all leave-one-out *p*-values remained below 0.05. After excluding the four flagged observations, the GenAI coefficient also remained positive and statistically significant (*B* = 3.055; 95% CI, 2.332 to 3.777; *p* < 0.001) (Table 2; Supplementary Table S6; Supplementary Figure S2).

### Patient perspective: GenAI utilization, communication satisfaction, and perceived empathy

**Figure 3:**
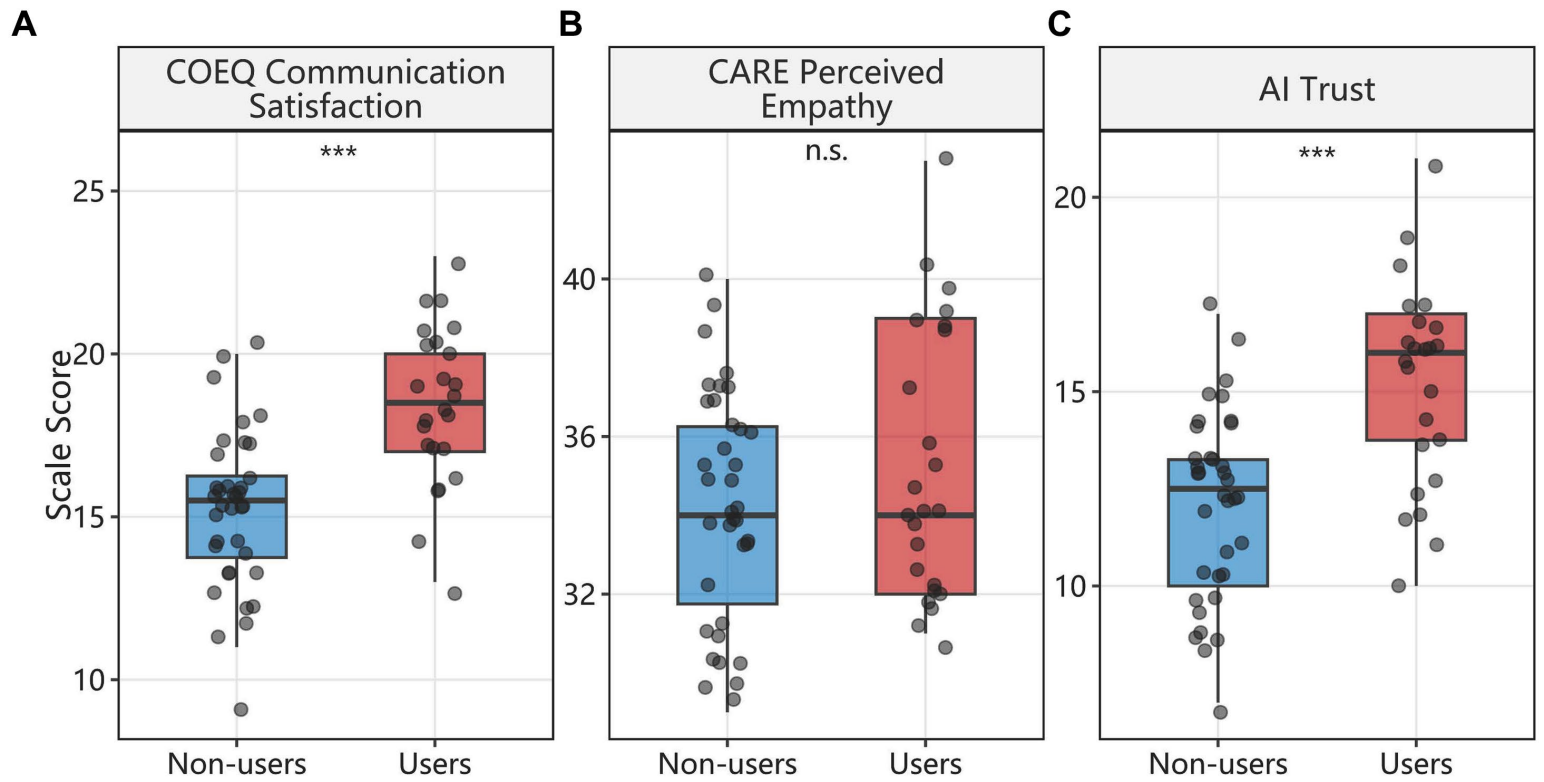
Boxplots comparing GenAI non-users (blue) and users (red) on **(A)** COEQ communication satisfaction, **(B)** CARE perceived empathy, and **(C)** AI trust. Cohen’s *d* effect sizes are reported above each panel (*p* < 0.001; n.s., not significant).

Compared with non-users, patients who had used GenAI to obtain skin health information within the preceding 4 weeks reported significantly higher communication satisfaction scores, as measured by the COEQ (18.46 ± 2.48 vs. 15.17 ± 2.43, *p* < 0.001). However, no statistically significant difference was observed between the two groups regarding perceived empathy scores on the CARE scale (35.25 ± 3.43 vs. 34.22 ± 2.95, *p* = 0.220). Furthermore, levels of trust in AI were significantly higher among users than non-users (15.21 ± 2.62 vs. 12.06 ± 2.34, *p* < 0.001) (Table 3A; Figure 3; Supplementary Table S7).

**Table 3A:** Patient-reported outcomes by recent GenAI use.

Outcome	Users M ± SD	Non-users M ± SD	*t*(df)	*p*-value	Cohen’s *d*
COEQ communication satisfaction	18.46 ± 2.48	15.17 ± 2.43	5.092 (58)	<0.001	1.342
CARE perceived empathy	35.25 ± 3.43	34.22 ± 2.95	1.239 (58)	0.220	0.327
AI trust	15.21 ± 2.62	12.06 ± 2.34	4.871 (58)	<0.001	1.284

In the multivariable linear regression analysis with communication satisfaction as the dependent variable, recent GenAI utilization remained associated with higher satisfaction levels after adjusting for age, educational attainment, hospital affiliation, and disease type (*B* = 2.866; SE = 0.765; 95% CI, 1.329–4.402; *p* < 0.001). This adjusted association remained statistically significant (*t* = 3.748, *p* < 0.001) (Table 3B; Supplementary Figure S3).

**Table 3B:** Multivariable linear regression analysis for COEQ communication satisfaction.

Predictor	*B* (SE)	95% CI	*p*-value
Recent GenAI use (yes vs. no)	2.866 (0.765)	1.329 to 4.402	<0.001
Age, years	−0.010 (0.033)	−0.075 to 0.056	0.770
High school/technical secondary (vs junior high or below)	1.522 (1.075)	−0.637 to 3.682	0.163
Bachelor’s degree (vs junior high or below)	−0.019 (1.052)	−2.134 to 2.095	0.986
Postgraduate or above (vs junior high or below)	−0.966 (1.089)	−3.155 to 1.223	0.380
Suining Central Hospital (vs FAHZZU)	0.362 (0.657)	−0.959 to 1.682	0.584
Infectious dermatoses (vs inflammatory)	0.025 (0.992)	−1.967 to 2.018	0.980
Neoplastic/pigmentary dermatoses (vs inflammatory)	−0.177 (1.019)	−2.225 to 1.872	0.863
Cosmetic-related dermatoses (vs inflammatory)	0.891 (0.952)	−1.021 to 2.804	0.354
Other dermatoses (vs inflammatory)	−1.341 (1.065)	−3.481 to 0.800	0.214

Recent GenAI use refers to patient self-reported use during the previous 4 weeks. The regression model was adjusted for age, educational attainment, hospital affiliation, and dermatologic disease category.

### Standardized case assessment: the impact of AI assistance on objective communication quality

Evaluation of 80 records from 20 physicians across two clinical scenarios (acne vulgaris and chronic eczema) and two preparation conditions confirmed that each physician completed all four condition-by-case combinations. Inter-rater reliability for standardized case scoring was good across all domains, with single-measure absolute-agreement ICCs ranging from 0.848 to 0.991 (Supplementary Table S3).

**Figure 4:**
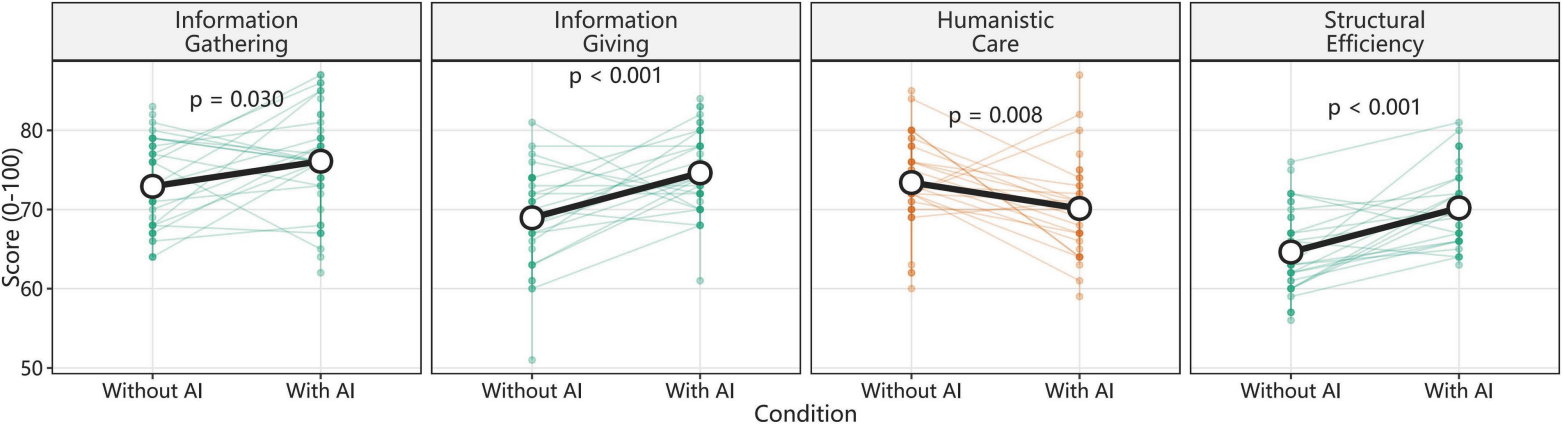
Paired comparisons of physician performance scores (0–100) across four communication domains between the AI and non-AI conditions. Thin lines connect individual paired observations; thick lines with open circles indicate mean scores. *p*-values from paired-samples *t*-tests are reported for each domain.

Estimated marginal means from the primary linear mixed-effects models showed that the AI-assisted condition was associated with higher scores for information gathering (95% CI, 0.605 to 5.745; *p* = 0.016), information giving (95% CI, 3.432 to 7.918; *p* < 0.001), structural efficiency (95% CI, 3.751 to 7.399; *p* < 0.001), and total score (95% CI, 1.374 to 3.606; *p* < 0.001). Humanistic care exhibited a reduction in the AI-assisted condition in the primary model; however, this difference did not reach statistical significance (95% CI, −5.524 to −1.026; *p* = 0.145) (Table 4; Figure 4). No statistically significant AI-by-case interaction was observed for any outcome (interaction *p*-values ranged from 0.176 to 0.889), suggesting that the overall AI effects were not driven by a single clinical case type (Supplementary Table S8). Sensitivity linear mixed-effects models that additionally adjusted for encounter period, condition order, and case order yielded consistent estimates for information-related and efficiency outcomes. For humanistic care, a consistent negative effect of AI assistance was observed in the fully adjusted sensitivity model (*B* = −3.414, 95% CI −5.723 to −1.105; *p* = 0.004), suggesting model-dependent variability but a stable directional trend (Supplementary Table S9; Supplementary Figure S4).

**Table 4:** Estimated marginal means and AI-assisted versus no-AI contrasts for standardized case outcomes.

Outcome	No AI M ± SD	AI-assisted M ± SD	Mean difference ± SD (diff)	*t*(df)	*p*-value	Cohen’s d_*z*	LMM AI effect *B* (SE)	LMM AI *p*	Case *B p*	AI × Case *p*
Information gathering	72.92 ± 5.60	76.10 ± 6.67	3.175 ± 8.935	2.247 (39)	0.030	0.355	4.550 (1.819)	0.015	0.530	0.290
Information giving	68.95 ± 5.77	74.62 ± 5.03	5.675 ± 7.474	4.802 (39)	<0.001	0.759	6.600 (1.588)	<0.001	0.107	0.413
Humanistic care	73.40 ± 5.67	70.12 ± 5.49	−3.275 ± 7.445	−2.782 (39)	0.008	−0.440	−2.350 (1.592)	0.145	0.108	0.415
Structural efficiency	64.62 ± 5.09	70.20 ± 4.66	5.575 ± 6.051	5.827 (39)	<0.001	0.921	5.450 (1.291)	<0.001	0.847	0.892
Total score	70.41 ± 3.19	72.90 ± 3.74	2.490 ± 4.004	3.934 (39)	<0.001	0.622	3.235 (0.790)	<0.001	0.046	0.188

Values are based on 40 within-physician paired observations (20 physicians × 2 standardized cases). Mean differences are calculated as AI-assisted minus no-AI scores. Linear mixed-effects models included fixed effects for AI condition and case type with a random intercept for the physician. Primary inference is based on linear mixed-effects models; paired t-tests are shown for descriptive comparison only.

### Exploratory analysis: variation in GenAI utilization across disease categories

Exploratory chi-squared analysis showed that recent GenAI use varied significantly across dermatologic disease categories [chi-square(4)=11.472, *p* = 0.022]. Patients with inflammatory dermatoses exhibited the highest utilization rate (59.1%, 13/22), followed by cosmetic-related dermatoses (50.0%, 5/10) and neoplastic/pigmentary dermatoses (40.0%, 4/10), whereas no use was reported among patients with infectious dermatoses (0/11). This analysis was exploratory and should not be interpreted as evidence of disease-specific causal preference (Supplementary Table S10; Supplementary Figure S5). Taken together, humanistic care showed model-dependent variability, with a non-significant effect in the primary model and a consistent negative association in fully adjusted sensitivity analyses.

## Discussion

**Figure 5:**
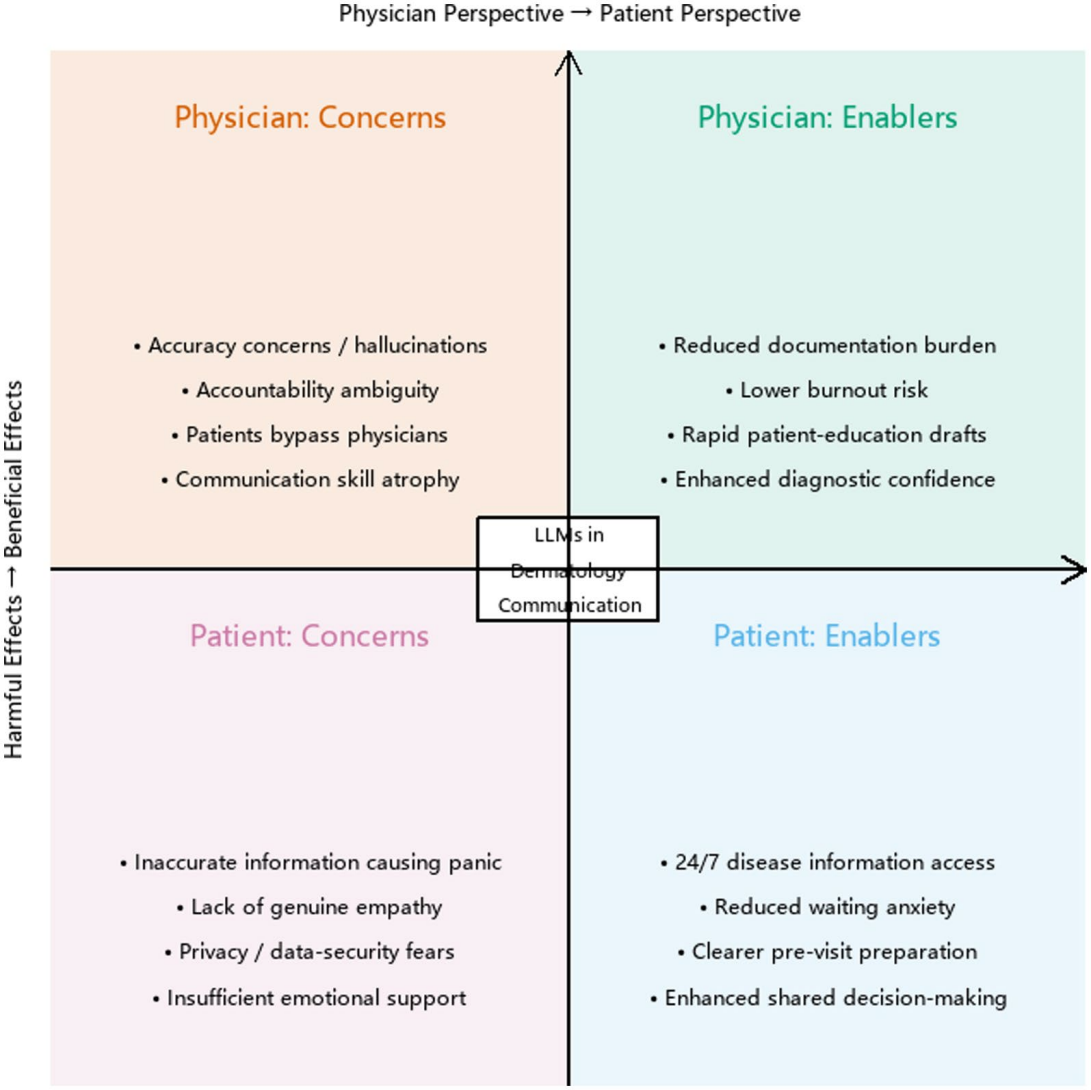
Conceptual framework of the double-edged effects of GenAI in dermatology communication.

This study provides multi-layered exploratory evidence from physicians, patients, and standardized case assessments, demonstrating that GenAI functions as a double-edged tool within dermatological communication. Across the three components, GenAI was most consistently associated with information synthesis, communicative efficiency, and pre-consultation preparation, whereas empathy and humanistic care did not improve in parallel. This pattern is consistent with prior dermatology and digital health studies indicating that AI is more acceptable and clinically useful as an adjunct to clinicians than as an autonomous substitute for physician judgment and relational communication (5, 6, 14–16). The relationships among these potential benefits and risks are summarized in the conceptual framework in Figure 5.

Regarding the physician perspective, increased use frequency was associated with lower emotional exhaustion and higher communication self-efficacy. The adjusted PCSES association was maintained across the primary model, a parsimonious model, leave-one-out refitting, and exclusion of observations flagged by influence diagnostics. These findings align with literature suggesting that AI tools may reduce repetitive explanation, documentation burden, and information restructuring. However, the small and unbalanced physician sample, particularly the frequent use subgroup of only three physicians, indicates that these associations should be interpreted as hypothesis-generating rather than as causal evidence that GenAI improves physician wellbeing or communication self-efficacy.

From the patient perspective, GenAI users reported higher communication satisfaction but did not perceive a corresponding increase in empathy. This finding is compatible with studies showing that AI-assisted health information tools can improve preparedness, question organization, and perceived access to information, while patient-provider relational trust and empathy remain dependent on the clinician’s in-person behavior. Platform, purpose, and frequency heterogeneity among patient GenAI users may also have influenced the observed association (17–19).

Our standardized case assessments further clarify this distinction. The AI-assisted preparation condition was associated with higher information gathering, information giving, structural efficiency, and total scores, and these contrasts were not materially changed after adjusting for encounter period, condition order, and case order. However, humanistic care scores were lower under the AI-assisted condition. One plausible explanation is that physicians who rely on model-generated wording may deliver more complete and organized information while becoming less spontaneous, less emotionally attuned, or more template-driven. Dermatology consultations often involve visible lesions, stigma, chronic recurrence, cosmetic concerns, and privacy-sensitive issues; therefore, communication quality depends not only on accurate information but also on individualized reassurance and emotional recognition.

The apparent divergence between higher patient satisfaction and lower standardized case humanistic care is important. In real outpatient settings, patients may place greater value on informational clarity, reduced uncertainty, and improved visit preparedness when reporting satisfaction and may overlook moderate losses in empathic nuance when their practical questions are answered. By contrast, standardized case scoring is designed to detect subtle deficits in emotional response, privacy sensitivity, and patient-centered pacing. Thus, the two levels of analysis may be complementary rather than contradictory: GenAI may improve information-related satisfaction while leaving relational communication vulnerable. Since this study was exploratory and used simulated encounters, this interpretation should be viewed as a plausible signal rather than the definitive evidence of benefit or harm.

This study carries several practical implications. First, GenAI is best suited for integration into patient education, drafting medication instructions, follow-up reminders, and pre-processing outpatient messages, rather than acting as an independent communicative entity (20, 21). Second, healthcare institutions deploying these tools should integrate performance metrics, auditing mechanisms, source transparency, and specialty-specific constraints into the workflow to mitigate model hallucinations and misinformation (22, 23). Third, professional training should focus not only on technical AI proficiency but also on techniques to maintain individualized and empathetic expression within an AI-assisted framework (24, 25).

Several limitations should be noted. First, the cross-sectional physician and patient components preclude strict causal inference. Second, the physician sample was small, the frequent use subgroup included only three physicians, and the physician model had a high predictor-to-sample ratio, increasing uncertainty despite influence and leave-one-out sensitivity analyses. Third, although the standardized case sequence was randomized and the sensitivity models were adjusted for period, condition order, and case order, all four encounters were completed consecutively on the same day; residual learning, fatigue, or carryover effects cannot be completely excluded. Fourth, the study was conducted at two tertiary hospitals in China, where AI attitudes, digital literacy, outpatient workload, and physician–patient communication norms may differ from those in primary care, private practice, rural settings, or other healthcare systems. Fifth, standardized cases improve internal comparability but cannot entirely reproduce real outpatient encounters. Sixth, patient-side GenAI use was self-reported and heterogeneous across platforms, purposes, and frequency, which may introduce recall bias and residual confounding. Finally, assessor-level reliability was confirmed using authentic independent ratings, with all ICC (A,1) values exceeding 0.75. Taken together, these findings should be interpreted alongside broader evidence on AI-assisted medicine and AI-driven digital scribe systems, which suggests that AI tools may reduce documentation burden and improve workflow efficiency, while implementation should still be evaluated against clinician experience, encounter quality, and electronic-health-record-related burnout (26–29).

In conclusion, GenAI in dermatology is neither a singular “panacea” nor a “threat” but a double-edged supportive tool. In this exploratory study, AI-assisted preparation was associated with stronger information organization and structural efficiency, while gains did not extend to humanistic care. These findings support supervised, carefully calibrated use of GenAI as an adjunct to clinical communication rather than as a replacement for physician judgment or relational care.

## Data Availability

The original contributions presented in the study are included in the article/Supplementary material, further inquiries can be directed to the corresponding author.
