# Supplementary figures and images for: The double-edged sword of generative AI in dermatology: a multi-component cross-sectional study on physician burnout, patient satisfaction, and communication quality
Source: Front Med (Lausanne). 2026 Jul 8;13:1875075. doi: 10.3389/fmed.2026.1875075 (PMC13388157; doi:10.3389/fmed.2026.1875075)

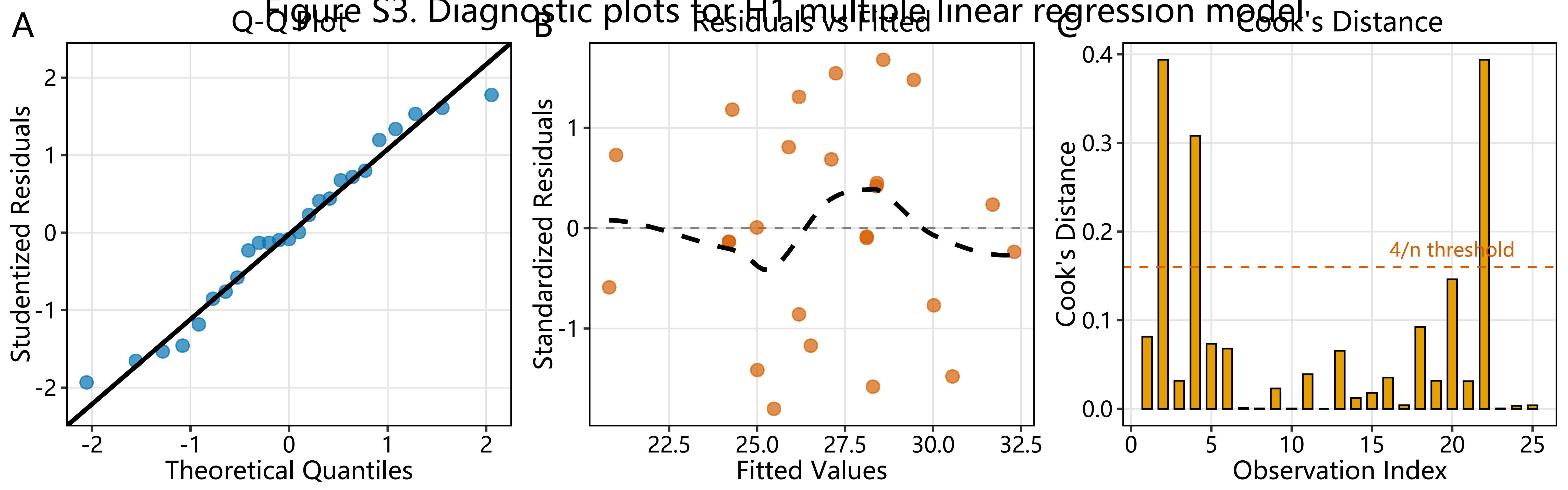

Supplement: Supplementary file 1 [file Image_1.JPEG]

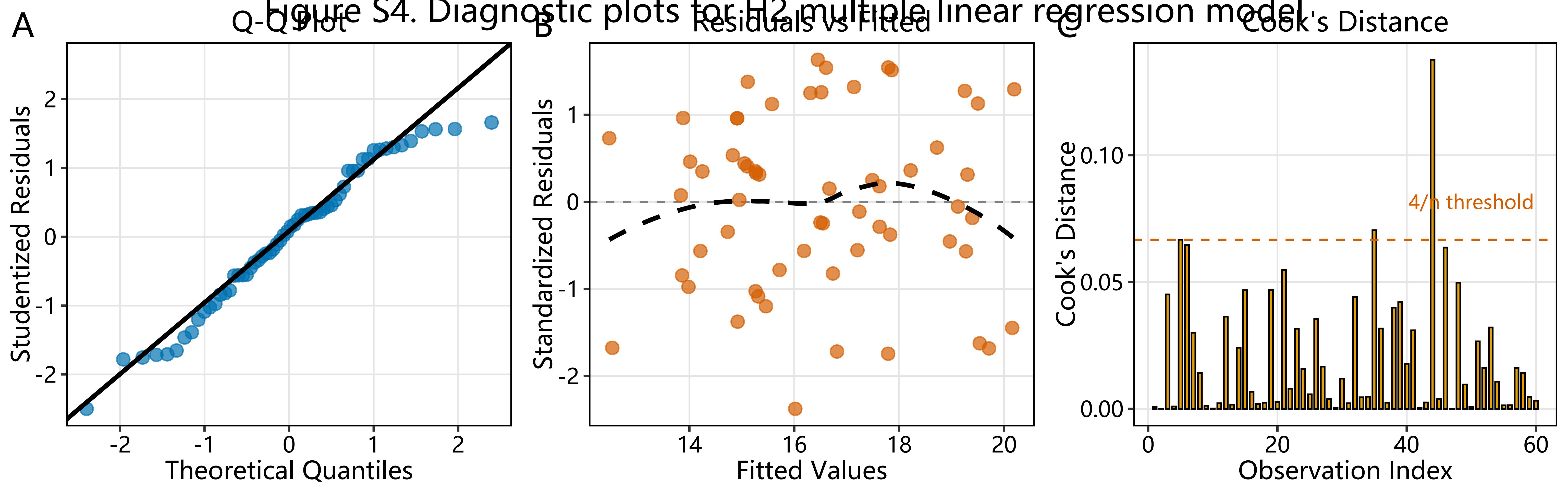

Supplement: Supplementary file 2 [file Image_2.JPEG]

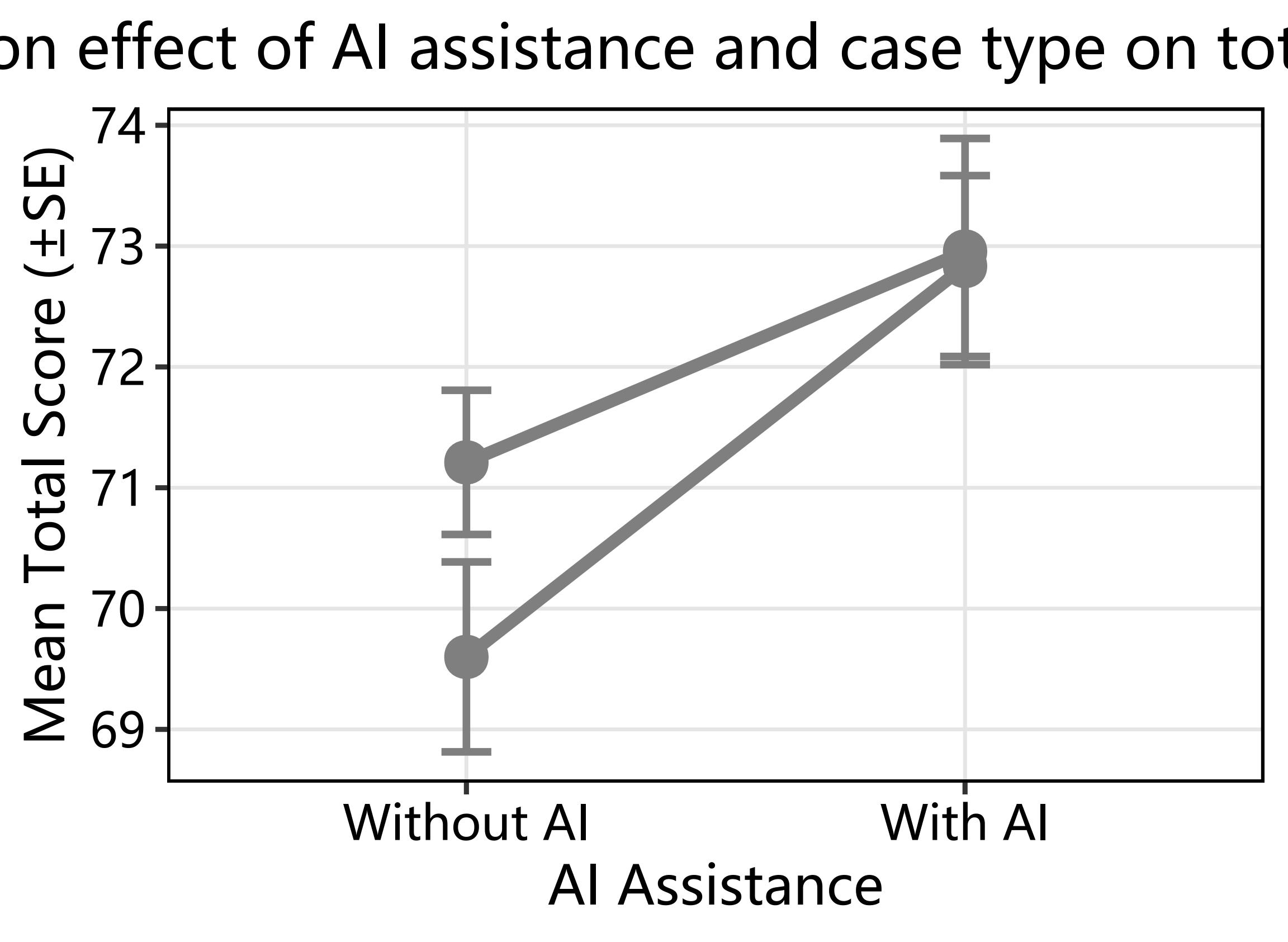

Supplement: Supplementary file 3 [file Image_3.JPEG]

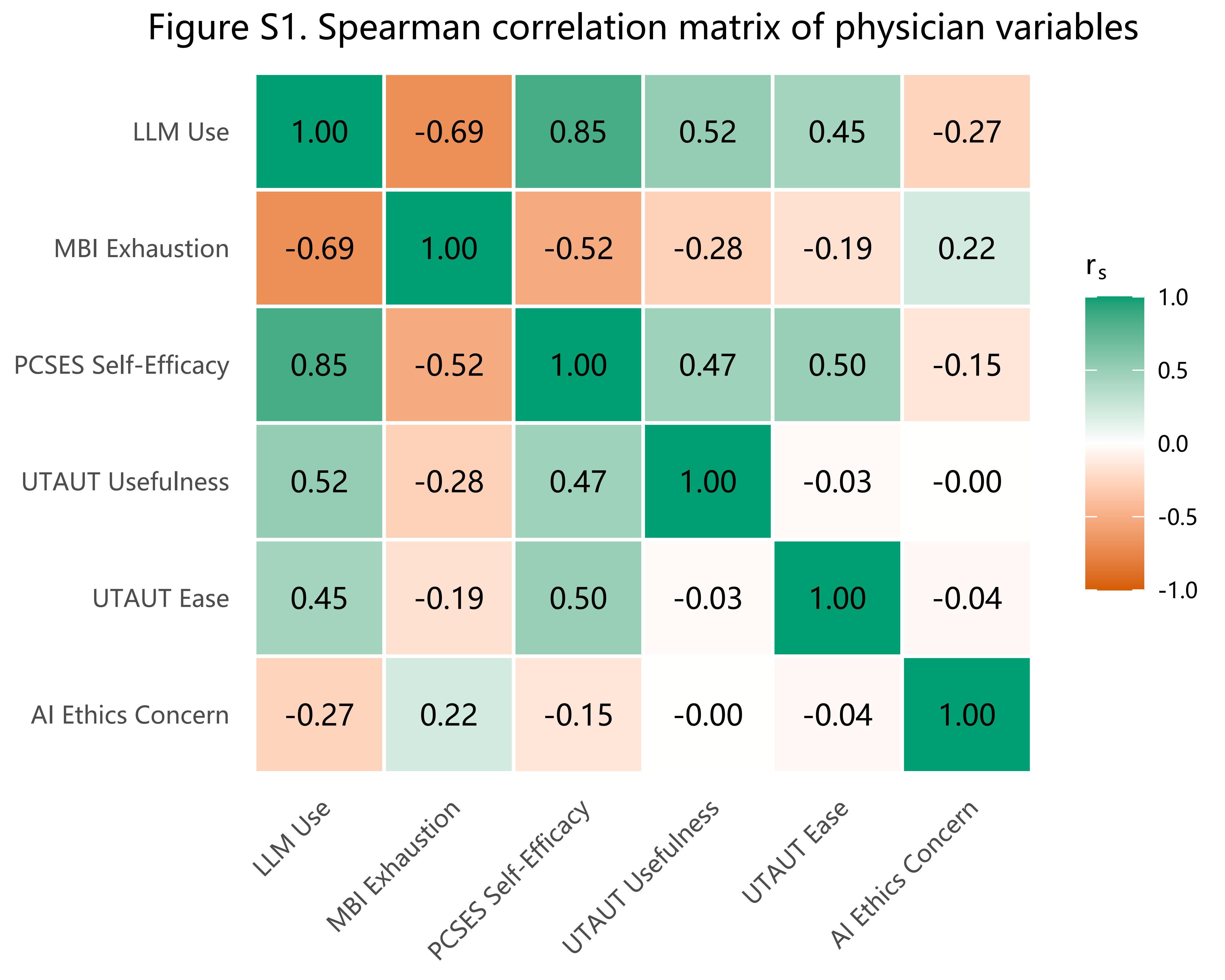

Supplement: Supplementary file 4 [file Image_4.JPEG]

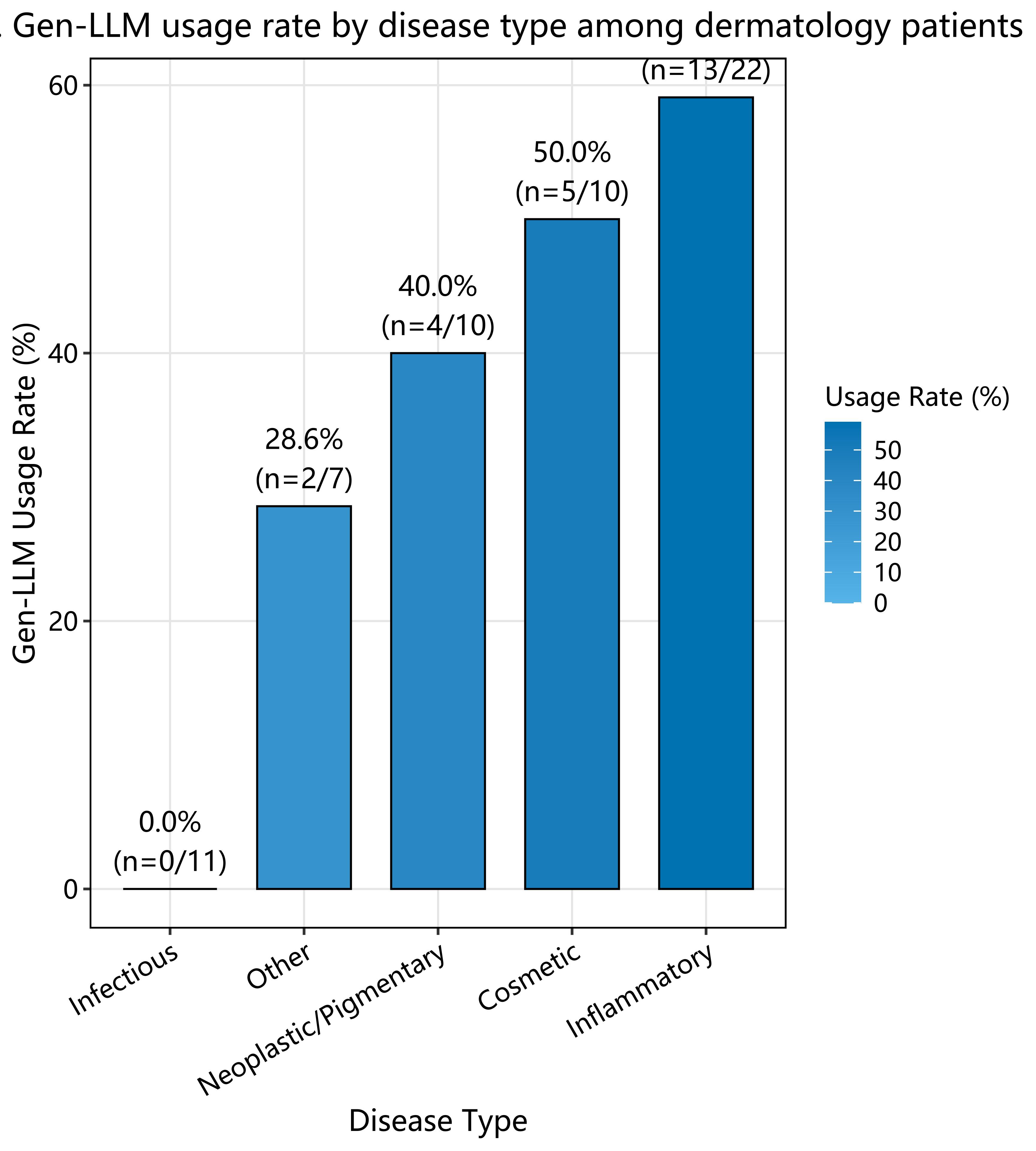

Supplement: Supplementary file 5 [file Image_5.JPEG]
