# Supplementary material for: The double-edged sword of generative AI in dermatology: a multi-component cross-sectional study on physician burnout, patient satisfaction, and communication quality
Source: Front Med (Lausanne). 2026 Jul 8;13:1875075. doi: 10.3389/fmed.2026.1875075 (PMC13388157; doi:10.3389/fmed.2026.1875075)
Supplement: Supplementary file 6 [file Table_1.DOCX]

# Supplementary Tables

## Supplementary Table S1. Standardized-case communication scoring rubric

| **Domain** | **Score range** | **Anchor description** |
| --- | --- | --- |
| Humanistic care | 90-100 | Actively recognizes patient emotion such as anxiety or embarrassment, uses empathic language, provides emotional support, respects privacy concerns, and maintains patient-centered pacing. |
| Humanistic care | 80-89 | Basic politeness with greeting and closing; emotional response is present but relatively passive; communication mainly focuses on information transfer. |
| Humanistic care | 60-79 | Mechanical question-answer style, limited eye contact or equivalent nonverbal engagement, and a task-completion orientation. |
| Humanistic care | 0-59 | Interrupts patient narrative, ignores patient questions, displays coldness, or uses inappropriate wording. |
| Information gathering | 0-100 | Completeness, sequence, and relevance of history-taking and patient concern elicitation. |
| Information provision | 0-100 | Accuracy, completeness, clarity, and patient accessibility of explanations and health education. |
| Structural efficiency | 0-100 | Logical organization, time management, signposting, and avoidance of unnecessary repetition. |
| Overall score | 0-100 | Global communication performance integrating task completion, patient-centeredness, and clinical communication quality. |

## Supplementary Table S2. AI-assisted pre-consultation preparation prompt and workflow

| **Item** | **Content** |
| --- | --- |
| Model | DeepSeek-R1 |
| Timing | Pre-consultation preparation only; up to 5 minutes before entering the standardized-patient encounter. |
| Researcher-provided case summary | Brief history summary consistent with the standardized-patient script. |
| Permitted physician behavior | Physicians could freely ask questions, request explanation drafts, ask for patient-education phrasing, and adopt, modify, or ignore AI suggestions. |
| Researcher intervention | Researchers did not intervene in query content or use strategy; backend query counts and query contents were recorded. |
| Representative prompt template | You are assisting a dermatologist preparing for a standardized patient consultation. Based on the following brief history, list key communication points, patient-friendly explanations, likely patient concerns, and reminders for empathic communication. Brief history: [case summary]. |

## Supplementary Table S3. Inter-rater reliability results for standardized-case ratings

| **Domain** | **N records** | **Primary raters included** | **Adjudicated by Assessor C, n** | **Score discrepancy >15, n** | **ICC(A,1) single-measure absolute agreement** | **ICC(A,k) average-measure absolute agreement** | **Interpretation** |
| --- | --- | --- | --- | --- | --- | --- | --- |
| Information gathering | 80 | Assessor A and Assessor B | 1 | 1 | 0.896 | 0.945 | good inter-rater reliability (>0.75) |
| Information provision | 80 | Assessor A and Assessor B | 3 | 3 | 0.848 | 0.918 | good inter-rater reliability (>0.75) |
| Humanistic care | 80 | Assessor A and Assessor B | 0 | 0 | 0.956 | 0.978 | good inter-rater reliability (>0.75) |
| Structural efficiency | 80 | Assessor A and Assessor B | 1 | 1 | 0.916 | 0.956 | good inter-rater reliability (>0.75) |
| Total score | 80 | Assessor A and Assessor B | 0 | 0 | 0.991 | 0.995 | good inter-rater reliability (>0.75) |

## Supplementary Table S4. Physician descriptive statistics

| **Variable** | **n** | **Mean ± SD** | **Median (IQR)** | **Range** |
| --- | --- | --- | --- | --- |
| Age, years | 25 | 37.72 ± 7.13 | 36.00 (33.00 to 42.00) | 28.00 to 53.00 |
| Work experience, years | 25 | 8.16 ± 5.68 | 7.00 (4.00 to 10.00) | 1.00 to 21.00 |
| GenAI use frequency (1-4) | 25 | 2.60 ± 0.82 | 3.00 (2.00 to 3.00) | 1.00 to 4.00 |
| MBI-GS emotional exhaustion | 25 | 12.16 ± 1.99 | 12.00 (11.00 to 13.00) | 9.00 to 17.00 |
| PCSES communication self-efficacy | 25 | 26.92 ± 3.19 | 28.00 (25.00 to 29.00) | 20.00 to 32.00 |
| UTAUT perceived usefulness | 25 | 13.64 ± 2.00 | 14.00 (12.00 to 15.00) | 10.00 to 17.00 |
| UTAUT perceived ease of use | 25 | 14.64 ± 2.00 | 15.00 (13.00 to 16.00) | 10.00 to 18.00 |
| UTAUT intention to use | 25 | 12.16 ± 1.95 | 12.00 (11.00 to 14.00) | 9.00 to 16.00 |
| UTAUT use behavior | 25 | 10.40 ± 2.06 | 10.00 (9.00 to 12.00) | 7.00 to 14.00 |
| AI ethics: accuracy concern | 25 | 9.96 ± 1.49 | 10.00 (9.00 to 11.00) | 7.00 to 12.00 |
| AI ethics: privacy concern | 25 | 8.80 ± 1.19 | 9.00 (8.00 to 10.00) | 7.00 to 11.00 |
| AI ethics: responsibility concern | 25 | 10.36 ± 1.44 | 10.00 (9.00 to 11.00) | 7.00 to 13.00 |
| AI ethics: doctor-patient relationship concern | 25 | 9.64 ± 2.00 | 9.00 (8.00 to 11.00) | 6.00 to 14.00 |
| AI ethics: total concern | 25 | 38.76 ± 2.80 | 39.00 (37.00 to 41.00) | 35.00 to 44.00 |

## Supplementary Table S5. Variance inflation factors for the physician regression model

| **Predictor** | **R-squared from auxiliary model** | **VIF** | **Interpretation** |
| --- | --- | --- | --- |
| GenAI use frequency | 0.325 | 1.48 | low collinearity |
| Attending physician (vs resident) | 0.712 | 3.47 | low collinearity |
| Associate chief physician (vs resident) | 0.848 | 6.58 | moderate collinearity |
| Chief physician (vs resident) | 0.859 | 7.09 | moderate collinearity |
| Work experience, years | 0.873 | 7.9 | moderate collinearity |
| Suining People's Hospital (vs FAHZZU) | 0.027 | 1.03 | low collinearity |

## Supplementary Table S6. Influence diagnostics and leave-one-out sensitivity analyses for the physician regression model

| **Physician ID** | **Cook's D** | **Leverage** | **Studentized residual** | **DFBETA for GenAI use frequency** | **Influential by prespecified thresholds** | **Reason** | **Leave-one-out GenAI B** | **Leave-one-out 95% CI** | **Leave-one-out P value** |
| --- | --- | --- | --- | --- | --- | --- | --- | --- | --- |
| D001 | 0.081 | 0.168 | 1.777 | 0.211 | No |  | 3.108 | 2.212 to 4.004 | <0.001 |
| D002 | 0.394 | 0.558 | 1.532 | 0.046 | Yes | Cook's D > 4/n | 3.183 | 2.274 to 4.091 | <0.001 |
| D003 | 0.032 | 0.231 | -0.853 | 0.23 | No |  | 3.092 | 2.110 to 4.075 | <0.001 |
| D004 | 0.308 | 0.475 | 1.611 | -0.193 | Yes | Cook's D > 4/n | 3.293 | 2.384 to 4.202 | <0.001 |
| D005 | 0.073 | 0.231 | 1.337 | -0.361 | No |  | 3.374 | 2.419 to 4.329 | <0.001 |
| D006 | 0.068 | 0.192 | -1.458 | 0.041 | No |  | 3.185 | 2.271 to 4.098 | <0.001 |
| D007 | 0.001 | 0.472 | -0.096 | 0.011 | No |  | 3.199 | 2.224 to 4.173 | <0.001 |
| D008 | 0.0 | 0.149 | -0.131 | -0.002 | No |  | 3.205 | 2.237 to 4.173 | <0.001 |
| D009 | 0.023 | 0.232 | 0.719 | -0.237 | No |  | 3.319 | 2.315 to 4.324 | <0.001 |
| D010 | 0.0 | 0.286 | -0.082 | -0.027 | No |  | 3.217 | 2.198 to 4.237 | <0.001 |
| D011 | 0.039 | 0.163 | 1.196 | 0.021 | No |  | 3.194 | 2.264 to 4.125 | <0.001 |
| D012 | 0.0 | 0.219 | 0.007 | -0.002 | No |  | 3.205 | 2.212 to 4.197 | <0.001 |
| D013 | 0.066 | 0.155 | -1.654 | -0.193 | No |  | 3.292 | 2.387 to 4.198 | <0.001 |
| D014 | 0.012 | 0.297 | 0.44 | 0.147 | No |  | 3.132 | 2.117 to 4.147 | <0.001 |
| D015 | 0.018 | 0.266 | -0.579 | 0.196 | No |  | 3.108 | 2.096 to 4.121 | <0.001 |
| D016 | 0.035 | 0.294 | -0.76 | -0.235 | No |  | 3.318 | 2.321 to 4.315 | <0.001 |
| D017 | 0.004 | 0.139 | 0.408 | 0.047 | No |  | 3.181 | 2.210 to 4.151 | <0.001 |
| D018 | 0.092 | 0.32 | -1.184 | 0.066 | No |  | 3.173 | 2.240 to 4.105 | <0.001 |
| D019 | 0.032 | 0.321 | 0.675 | -0.036 | No |  | 3.221 | 2.264 to 4.179 | <0.001 |
| D020 | 0.146 | 0.24 | -1.932 | 0.434 | Yes | \|DFBETA GenAI\| > 2/sqrt(n) | 3.01 | 2.111 to 3.909 | <0.001 |
| D021 | 0.031 | 0.25 | 0.8 | -0.22 | No |  | 3.311 | 2.324 to 4.297 | <0.001 |
| D022 | 0.394 | 0.558 | -1.532 | 0.046 | Yes | Cook's D > 4/n | 3.183 | 2.274 to 4.091 | <0.001 |
| D023 | 0.0 | 0.149 | -0.131 | -0.002 | No |  | 3.205 | 2.237 to 4.173 | <0.001 |
| D024 | 0.003 | 0.302 | 0.229 | 0.113 | No |  | 3.148 | 2.070 to 4.227 | <0.001 |
| D025 | 0.004 | 0.331 | -0.23 | -0.104 | No |  | 3.255 | 2.193 to 4.317 | <0.001 |

## Supplementary Table S7. Patient descriptive statistics stratified by hospital and GenAI use

| **Panel / subgroup** | **n** | **Age, years** | **eHEALS** | **COEQ** | **CARE** | **AI trust** | **GenAI users, n (%)** | **Privacy-sensitive, n (%)** |
| --- | --- | --- | --- | --- | --- | --- | --- | --- |
| Panel A. Stratified by hospital |  |  |  |  |  |  |  |  |
| First Affiliated Hospital of Zhengzhou University | 35 | 40.94 ± 10.65 | 22.57 ± 4.08 | 16.46 ± 3.23 | 34.77 ± 3.50 | 13.46 ± 3.07 | 16 (45.7) | 4 (11.4) |
| Suining People's Hospital | 25 | 42.52 ± 10.57 | 22.20 ± 4.43 | 16.52 ± 2.50 | 34.44 ± 2.68 | 13.12 ± 2.67 | 8 (32.0) | 4 (16.0) |
| Panel B. Stratified by dermatologic diagnosis |  |  |  |  |  |  |  |  |
| Inflammatory dermatoses | 22 | 42.50 ± 10.82 | 22.32 ± 4.64 | 16.91 ± 2.56 | 33.59 ± 2.74 | 13.91 ± 3.10 | 13 (59.1) | 1 (4.5) |
| Infectious dermatoses | 11 | 41.36 ± 6.55 | 22.45 ± 3.78 | 15.09 ± 1.81 | 33.18 ± 3.31 | 12.91 ± 2.63 | 0 (0.0) | 3 (27.3) |
| Neoplastic / pigmentary dermatoses | 10 | 38.50 ± 12.64 | 20.60 ± 3.78 | 17.10 ± 3.03 | 35.80 ± 2.78 | 13.50 ± 3.10 | 4 (40.0) | 0 (0.0) |
| Cosmetic-related dermatoses | 10 | 42.30 ± 13.06 | 24.10 ± 3.63 | 17.60 ± 3.13 | 35.40 ± 2.67 | 12.70 ± 1.70 | 5 (50.0) | 3 (30.0) |
| Other | 7 | 42.57 ± 9.88 | 22.86 ± 4.74 | 14.86 ± 4.14 | 37.43 ± 3.36 | 12.71 ± 3.95 | 2 (28.6) | 1 (14.3) |
| Panel C. Stratified by education |  |  |  |  |  |  |  |  |
| Junior high or below | 8 | 39.00 ± 10.03 | 18.62 ± 2.50 | 16.00 ± 2.78 | 35.75 ± 5.20 | 12.38 ± 2.67 | 3 (37.5) | 0 (0.0) |
| High school / technical secondary | 22 | 43.36 ± 11.82 | 20.45 ± 3.79 | 17.86 ± 2.98 | 35.64 ± 2.68 | 13.45 ± 2.99 | 11 (50.0) | 3 (13.6) |
| Bachelor's degree | 18 | 41.06 ± 11.12 | 23.72 ± 3.18 | 16.11 ± 2.00 | 34.44 ± 2.38 | 13.17 ± 3.29 | 6 (33.3) | 3 (16.7) |
| Postgraduate or above | 12 | 40.92 ± 7.98 | 26.58 ± 2.78 | 14.83 ± 3.27 | 32.33 ± 2.27 | 13.92 ± 2.31 | 4 (33.3) | 2 (16.7) |

## Supplementary Table S8. Full fixed-effects estimates from standardized-case linear mixed-effects models

| **Outcome** | **Fixed effect** | **B** | **SE** | **df** | **95% CI** | **t** | **P value** |
| --- | --- | --- | --- | --- | --- | --- | --- |
| Information gathering | Intercept (No-AI, acne) | 72.35 | 1.351 | 75.6 | 69.702 to 74.998 | 53.56 | <0.001 |
| Information gathering | AI-assisted vs No-AI within acne | 4.55 | 1.773 | 60.0 | 1.075 to 8.025 | 2.566 | 0.013 |
| Information gathering | Chronic eczema vs acne within No-AI | 1.15 | 1.773 | 60.0 | -2.325 to 4.625 | 0.649 | 0.519 |
| Information gathering | AI x case interaction | -2.75 | 2.508 | 60.0 | -7.665 to 2.165 | -1.097 | 0.277 |
| Information giving | Intercept (No-AI, acne) | 67.65 | 1.176 | 75.9 | 65.345 to 69.955 | 57.535 | <0.001 |
| Information giving | AI-assisted vs No-AI within acne | 6.6 | 1.548 | 60.0 | 3.567 to 9.633 | 4.265 | <0.001 |
| Information giving | Chronic eczema vs acne within No-AI | 2.6 | 1.548 | 60.0 | -0.433 to 5.633 | 1.68 | 0.098 |
| Information giving | AI x case interaction | -1.85 | 2.189 | 60.0 | -6.139 to 2.439 | -0.845 | 0.401 |
| Humanistic care | Intercept (No-AI, acne) | 72.1 | 1.214 | 72.7 | 69.721 to 74.479 | 59.407 | <0.001 |
| Humanistic care | AI-assisted vs No-AI within acne | -2.35 | 1.551 | 60.0 | -5.391 to 0.691 | -1.515 | 0.135 |
| Humanistic care | Chronic eczema vs acne within No-AI | 2.6 | 1.551 | 60.0 | -0.441 to 5.641 | 1.676 | 0.099 |
| Humanistic care | AI x case interaction | -1.85 | 2.194 | 60.0 | -6.150 to 2.450 | -0.843 | 0.402 |
| Structural efficiency | Intercept (No-AI, acne) | 64.5 | 1.076 | 61.5 | 62.391 to 66.609 | 59.941 | <0.001 |
| Structural efficiency | AI-assisted vs No-AI within acne | 5.45 | 1.258 | 60.0 | 2.984 to 7.916 | 4.331 | <0.001 |
| Structural efficiency | Chronic eczema vs acne within No-AI | 0.25 | 1.258 | 60.0 | -2.216 to 2.716 | 0.199 | 0.843 |
| Structural efficiency | AI x case interaction | 0.25 | 1.78 | 60.0 | -3.238 to 3.738 | 0.14 | 0.889 |
| Total score | Intercept (No-AI, acne) | 69.6 | 0.757 | 47.1 | 68.117 to 71.083 | 91.989 | <0.001 |
| Total score | AI-assisted vs No-AI within acne | 3.235 | 0.77 | 60.0 | 1.726 to 4.744 | 4.203 | <0.001 |
| Total score | Chronic eczema vs acne within No-AI | 1.61 | 0.77 | 60.0 | 0.101 to 3.119 | 2.092 | 0.041 |
| Total score | AI x case interaction | -1.49 | 1.089 | 60.0 | -3.623 to 0.643 | -1.369 | 0.176 |

## Supplementary Table S9. Case-specific AI contrasts and order/period-adjusted sensitivity analyses

| **Outcome** | **Contrast** | **B** | **SE** | **df** | **95% CI** | **t** | **P value** |
| --- | --- | --- | --- | --- | --- | --- | --- |
| Information gathering | AI-assisted vs No-AI within Acne vulgaris | 4.55 | 1.819 | 63.2 | 0.915 to 8.185 | 2.501 | 0.015 |
| Information gathering | AI-assisted vs No-AI within Chronic eczema | 1.8 | 1.819 | 63.2 | -1.835 to 5.435 | 0.989 | 0.326 |
| Information gathering | AI-by-case interaction | -2.75 | 2.508 | 60.0 | -7.665 to 2.165 | -1.097 | 0.277 |
| Information giving | AI-assisted vs No-AI within Acne vulgaris | 6.6 | 1.588 | 63.2 | 3.427 to 9.773 | 4.157 | <0.001 |
| Information giving | AI-assisted vs No-AI within Chronic eczema | 4.75 | 1.588 | 63.2 | 1.577 to 7.923 | 2.992 | 0.004 |
| Information giving | AI-by-case interaction | -1.85 | 2.189 | 60.0 | -6.139 to 2.439 | -0.845 | 0.401 |
| Humanistic care | AI-assisted vs No-AI within Acne vulgaris | -2.35 | 1.592 | 63.2 | -5.531 to 0.831 | -1.476 | 0.145 |
| Humanistic care | AI-assisted vs No-AI within Chronic eczema | -4.2 | 1.592 | 63.2 | -7.381 to -1.019 | -2.639 | 0.010 |
| Humanistic care | AI-by-case interaction | -1.85 | 2.194 | 60.0 | -6.150 to 2.450 | -0.843 | 0.402 |
| Structural efficiency | AI-assisted vs No-AI within Acne vulgaris | 5.45 | 1.291 | 63.2 | 2.870 to 8.030 | 4.222 | <0.001 |
| Structural efficiency | AI-assisted vs No-AI within Chronic eczema | 5.7 | 1.291 | 63.2 | 3.120 to 8.280 | 4.415 | <0.001 |
| Structural efficiency | AI-by-case interaction | 0.25 | 1.78 | 60.0 | -3.238 to 3.738 | 0.14 | 0.889 |
| Total score | AI-assisted vs No-AI within Acne vulgaris | 3.235 | 0.79 | 63.2 | 1.657 to 4.813 | 4.097 | <0.001 |
| Total score | AI-assisted vs No-AI within Chronic eczema | 1.745 | 0.79 | 63.2 | 0.167 to 3.323 | 2.21 | 0.031 |
| Total score | AI-by-case interaction | -1.49 | 1.089 | 60.0 | -3.623 to 0.643 | -1.369 | 0.176 |
| Information gathering | Overall marginal AI-assisted vs No-AI adjusted for period, condition order, and case order | 2.838 | 1.304 | 67.3 | 0.235 to 5.441 | 2.176 | 0.033 |
| Information giving | Overall marginal AI-assisted vs No-AI adjusted for period, condition order, and case order | 5.309 | 1.131 | 64.3 | 3.049 to 7.569 | 4.692 | <0.001 |
| Humanistic care | Overall marginal AI-assisted vs No-AI adjusted for period, condition order, and case order | -3.414 | 1.156 | 64.3 | -5.723 to -1.105 | -2.953 | 0.004 |
| Structural efficiency | Overall marginal AI-assisted vs No-AI adjusted for period, condition order, and case order | 5.439 | 0.936 | 64.3 | 3.569 to 7.309 | 5.809 | <0.001 |
| Total score | Overall marginal AI-assisted vs No-AI adjusted for period, condition order, and case order | 2.209 | 0.545 | 64.3 | 1.121 to 3.298 | 4.054 | <0.001 |

## Supplementary Table S10. Exploratory GenAI use by disease type among patients

| **Disease type** | **Total n** | **GenAI users, n** | **Use rate (%)** |
| --- | --- | --- | --- |
| Inflammatory dermatoses | 22 | 13 | 59.1 |
| Infectious dermatoses | 11 | 0 | 0.0 |
| Neoplastic / pigmentary dermatoses | 10 | 4 | 40.0 |
| Cosmetic-related dermatoses | 10 | 5 | 50.0 |
| Other | 7 | 2 | 28.6 |

## Supplementary Table S11. Questionnaire instruments used in the physician and patient questionnaires

| **Instrument/indicator** | **Respondent group** | **Construct measured** | **Source/adaptation** | **Number of items** | **Score range** | **Score direction** | **Role in this study** |
| --- | --- | --- | --- | --- | --- | --- | --- |
| MBI-GS emotional exhaustion subscale | Physicians | Emotional exhaustion / occupational burnout symptom burden | Emotional-exhaustion subscale of the Maslach Burnout Inventory-General Survey (MBI-GS). | 5 | 5-25 | Higher scores indicate greater emotional exhaustion. | Physician-side outcome correlated with GenAI use frequency. |
| PCSES communication self-efficacy | Physicians | Physician confidence in clinical communication | Short-form Physician Communication Self-Efficacy Scale (PCSES) used for outpatient communication self-efficacy assessment. | 7 | 7-35 | Higher scores indicate stronger communication self-efficacy. | Physician-side outcome and dependent variable in the physician regression model. |
| UTAUT constructs | Physicians | Technology acceptance: perceived usefulness, perceived ease of use, intention to use, and use behavior | Four-domain adaptation of the Unified Theory of Acceptance and Use of Technology (UTAUT) framework for GenAI use in dermatology. | 16 total; 4 items per domain | 4-20 for each domain | Higher scores indicate stronger perceived usefulness, ease of use, intention, or use behavior. | Physician-side explanatory technology-acceptance indicators. |
| AI ethics concern | Physicians | Concerns about AI accuracy, privacy, responsibility, and doctor-patient relationship effects | Study-specific items developed with reference to AI ethics and clinician-AI perception literature and reviewed for dermatology relevance. | 12 total; 3 items per domain | 12-60 total; 3-15 per domain | Higher scores indicate stronger ethical concern. | Physician-side contextual indicator of AI-related concern. |
| eHEALS | Patients | eHealth literacy | eHealth Literacy Scale (eHEALS). | 8 | 8-40 | Higher scores indicate higher perceived eHealth literacy. | Patient-side descriptive variable and covariate/contextual factor. |
| COEQ communication satisfaction | Patients | Outpatient communication satisfaction | Communication domain of the Chinese Outpatient Experience Questionnaire (COEQ). | 5 | 5-25 | Higher scores indicate higher communication satisfaction. | Primary patient-side outcome and dependent variable in the patient regression model. |
| CARE perceived empathy | Patients | Patient-perceived clinician empathy | Consultation and Relational Empathy (CARE) measure, patient-rated short form. | 10 | 10-50 | Higher scores indicate stronger perceived empathy. | Patient-side relational communication outcome. |
| AI trust | Patients | Trust in AI-assisted health information and AI-supported communication | Study-specific/adapted items informed by healthcare AI trust literature and reviewed for dermatology relevance. | 5 | 5-25 | Higher scores indicate greater trust in AI. | Patient-side contextual outcome comparing recent GenAI users and non-users. |
